# Retraction: Construction and validation of nomogram model for endoscopic submucosal dissection in patients with early colorectal cancer based on intestinal microflora and clinical pathological parameters

**DOI:** 10.3389/fmed.2026.1867354

**Published:** 2026-05-04

**Authors:** Frontiers Editorial Office

The journal retracts the 30 May 2025 article cited above.

Following publication, concerns were raised regarding the validity of the data in the article. The authors failed to provide a satisfactory explanation during the investigation, which was conducted in accordance with Frontiers' policies.

This retraction was approved by the Chief Editors of Frontiers in Medicine and the Chief Executive Editor of Frontiers. The authors received a communication regarding the retraction and had a chance to respond. This communication has been recorded by the publisher.

